# Jellyfish from Fisheries By-Catches as a Sustainable Source of High-Value Compounds with Biotechnological Applications

**DOI:** 10.3390/md20040266

**Published:** 2022-04-14

**Authors:** Isabella D’Ambra, Louise Merquiol

**Affiliations:** Integrative Marine Ecology Department, Stazione Zoologica Anton Dohrn, Villa Comunale, 80121 Napoli, Italy; louise.merquiol@szn.it

**Keywords:** biochemical composition, food, nutraceuticals, collagen, toxins, scaffolds, anti-cancers, drug delivery, biomaterials, mucus, circular economy

## Abstract

The world’s population growth and consequent increased demand for food, energy and materials together with the decrease of some natural resources have highlighted the compelling need to use sustainably existing resources and find alternative sources to satisfy the needs of growing and longer-aging populations. In this review, we explore the potential use of a specific fisheries by-catch, jellyfish, as a sustainable source of high-value compounds. Jellyfish are often caught up with fish into fishing gear and nets, then sorted and discarded. Conversely, we suggest that this by-catch may be used to obtain food, nutraceutical products, collagen, toxins and fluorescent compounds to be used for biomedical applications and mucus for biomaterials. These applications are based on studies which indicate the feasibility of using jellyfish for biotechnology. Because jellyfish exhibit seasonal fluctuations in abundance, jellyfish by-catches likely follow the same pattern. Therefore, this resource may not be constantly available throughout the year, so the exploitation of the variable abundances needs to be optimized. Despite the lack of data about jellyfish by-catches, the high value of their compounds and their wide range of applications suggest that jellyfish by-catches are a resource which is discarded at present, but needs to be re-evaluated for exploitation within the context of a circular economy in the era of zero waste.

## 1. Introduction

“True jellyfish” include pelagic organisms belonging to three classes of the phylum Cnidaria (Hydrozoa, Scyphozoa and Cubozoa) [1]. Considered for a long time a nuisance because they damage fisheries gear and the fish caught within them, impair the proper functioning of power plants, sting swimmers along the coasts during normal, and have exceptionally high abundances (outbreaks) [2], jellyfish are a cosmopolitan and ancient component of the pelagic environment as they have survived different and often adverse environmental conditions since the Cambrian era [3,4] and have shown a high adaptive radiation by colonizing all marine environments [5]. Despite research efforts, it remains unclear whether jellyfish are increasing due to climate change and increased anthropogenic stressors, or if their abundance follows a cyclic pattern of which we are experiencing one of the high abundance phases [6]. Whatever is the case, high abundances of jellyfish are likely to occur with variable and potentially increasing frequency, and therefore managing them is an important task within the near future [2].

Jellyfish are actively fished in areas where they are exploited as a food or to produce collagen [7,8,9]. Despite the fact that the data need to be updated, China has the oldest and most active jellyfish fisheries because the Chinese have been consuming jellyfish directly as a food on a daily basis for centuries [10,11]. Likely as a consequence of the immigration of Chinese citizens, other eastern countries such as Japan, Malaysia, Taiwan and Singapore, where the consumption of jellyfish was not as deep-rooted as in China, started to actively fish jellyfish [7]. Similarly, the immigration of people from eastern into western countries, an increased export toward eastern countries, and the collapse of local traditional fisheries due to the decrease of fish stocks prompted jellyfish fisheries in western countries [8]. According to the latest estimates updated to 2016, jellyfish fisheries account for about 3% of the total fishery landings on a global scale [8].

Active jellyfish fisheries have not yet developed in Europe [12], apart from an isolated, very recent (since 2014) case in the United Kingdom [9]. Despite the doubtful identification of a scyphomedusa as a recipe ingredient from ancient Romans, jellyfish are not part of the traditional diet of northern and southern Europeans. Nevertheless, active fisheries targeting the rhizostome scyphomedusa *Rhizostoma octopus* started in 2014 within UK waters as a consequence of the exploitation of this species to extract a high-quality collagen used for biomedical applications [9].

Although jellyfish are commonly perceived as a nuisance, their uncontrolled removal from the pelagic environment may produce negative effects on the marine ecosystem. Jellyfish are blamed for competing with fish, including commercially relevant species, for plankton prey. While this trophic overlap has been observed to be partially occurring in some areas [13,14], to date it has never been demonstrated that jellyfish outcompete fish for prey. Conversely, jellyfish are preyed upon by a variety of economically and ecologically important predators, including fish, sea turtles, sharks and sea birds [15,16,17,18]. In particular, the relationship between jellyfish and fish larvae may be more complex than previously considered, as highlighted by recent studies [19,20]. Additionally, jellyfish transfer organic matter not only toward higher trophic levels of the food web but also to the oligotrophic sea bottom through “jelly-falls” [21,22]. Therefore, removing uncontrolled amounts of jellyfish from the environment may cause a cascading effect on the entire ecosystem and alter balances that have remained stable for ages [23]. A recent example of the concern raised by the uncontrolled removal of jellyfish was reported in the UK, where jellyfish started to be removed to be processed for commercial purposes [9]. The results of the study indicated that during the years with a low abundance of the scyphomedusa *Rhizostoma octopus*, the leatherback sea turtle *Dermochelys coricea*, which prey upon them, may be food limited due to the harvesting of the scyphomedusae [9].

As a component of the pelagic environment, jellyfish are often caught up into fishing nets and gear during regular fisheries activities. Fishermen accuse jellyfish by-catches of being a problem because fishing nets and caught fishes are often damaged by the massive abundance of jellyfish, and fishermen themselves are stung. Although these damages appear to be common within fisheries, a quantification of the economic losses due to jellyfish is difficult to make and only a few attempts have been made [24]. Likewise, an estimate of jellyfish by-catches is difficult at present. Fisheries by-catches are available through the Food and Agriculture Organization of United Nations (https://www.fao.org/3/T4890E/T4890E00.htm, (accessed on 10 April 2022)), but the data are only up-to-date until 2014. Additionally, within fisheries by-catches, fishermen usually do not account for jellyfish or eventually include them within invertebrates [7].

Most jellyfish follow a seasonal pattern of abundance, and large fluctuations are observed across years [6,25]. Nevertheless, this potential limitation due to the variable availability of jellyfish may be resolved using an appropriate management strategy on a local scale. Jellyfish by-catches from an area may converge to a local collecting and processing site. The fact that active fisheries of jellyfish in eastern countries, North and South America as well as the UK have survived the fluctuating abundance of jellyfish suggests that the available biomass of jellyfish is sufficient to sustain the request of the market over time. Additionally, management strategies will need to calibrate the need for biotechnological applications on the available biomass in order to make jellyfish by-catches sufficient to satisfy the request. The disposal of organic waste is costly; therefore, finding solutions to minimize these costs is highly needed to enhance the benefits for the environment and society.

In this review, we selected the lines of research which provide a solid background to develop the potential uses of jellyfish or well-established examples of exploitation in different biotechnological fields, ranging from nutraceuticals to biomedicals and biomaterials. We suggest that pursuing the exploitation of jellyfish by-catches is an effective approach to dispose of them and that this neglected source of high-value compounds should not remain unexploited, particularly within the context of a circular economy and zero waste politics.

## 2. Potential Uses of Jellyfish Collected as Fisheries By-Catches

### 2.1. Nutraceuticals (Including Direct Consumption as a Food)

As we mentioned above, jellyfish have been consumed as a food in eastern countries across their long cooking tradition [11]. At present, specific studies to determine the beneficial effects of jellyfish on human health through their direct consumption are lacking, and the benefits are associated to the overall higher-quality diet of eastern populations compared to western ones. Among these benefits, eastern populations appear to experience less arthritis, lower blood pressure values and less problems due to a lipid-rich diet [11]. Recent studies appear to support the relationship between jellyfish consumption and the above-mentioned benefits, with jellyfish suggested for the production of treatments for arthritis, hypertension and inflammatory processes [26,27,28]. However, these studies have not been followed by further data to support the suggested pharmaceutical applications.

Despite the lack of specific studies to support the healthiness of a diet including jellyfish, an analysis of the biochemical composition of the organisms belonging to jellyfish (hydromedusae, scyphomedusae and cubomedusae) highlights some common patterns (Table 1).

The biochemical composition varies across organisms due to natural variability. While the bell and oral arms often have similar biochemical compositions, the gonads have a remarkably higher content of lipids. The difference is correlated with physiological processes, particularly sexual maturity and egg production, when lipids are stored into the gonads to facilitate reproduction [60]. Additionally, diverse analytical methods may result in different biochemical content estimations [61]. Jellyfish are composed of a large amount of water (between 93.4% and 99.8%) and salts in relation to their organic content which can bias the quantification of organic matter and biomass [61]. Overall, the protein content is higher than the content of lipids and carbohydrates (Table 1). Although most species contain between 10% and 20% (DM) of proteins overall, *Rhizostoma pulmo* and *Rhopilema esculentum* contain an almost twofold percentage of proteins compared to the other species (Table 1). *R. esculentum* is commonly consumed as a food in eastern countries [11].

Within the reduced amount of lipids, omega-3 (ω-3) polyunsaturated fatty acids (PUFAs) dominate the fatty acid composition (Table 2). Omega-3 has multiple benefits for human health including anti-inflammatory, anti-hypertensive, anti-oxidant, anti-depressive, anti-aging and anti-arthritic activities [62]. While contemporary western diets are promoting the genesis of many diseases such as cancer and cardiovascular diseases as a consequence of the excessive amounts of omega-6 (ω-6) PUFAs and a very high ω-6/ω-3 ratio due to their dietary habits and lifestyle [63,64], increased levels of ω-3 (a low ω-6/ω-3 ratio) in the diet may help reduce the onset of these diseases [64]. Although jellyfish are not likely to be exploited as a source of fatty acid integrators given their low content compared to other marine organisms [65], their biochemical composition makes them a healthy food to be included in the dietary composition of western countries, as suggested by recent studies [66].

In addition to healthiness due to biochemical composition, an increasing number of studies focused on Mediterranean scyphomedusae have detected their anti-oxidant effect, which supports their direct consumption as a food, but may also find a large application in nutraceuticals. These studies are related mostly to the scyphomedusae belonging to the order Rhizostomeae, the same order in which the scyphomedusae is consumed as a food in eastern countries, and include *Rhizostoma luteum* [55], *Rhizostoma pulmo* [75], *Cotylorhiza tuberculata* [43] and *Cassiopea andromeda* [52], but also the Semaeostomeae *Aurelia coerulea* [42].

Despite the fact that jellyfish have been consumed as a food in eastern countries for centuries, their processing for this purpose may need improvements. Traditional processing of jellyfish for human consumption includes the addition of aluminium salts in different amounts to reduce the water content and allow the transition from a gelatinous consistency into a crispy texture. Aluminium salt-based processing has been exported to the Americas together with the active jellyfish fisheries [8]. However, the residual aluminium in jellyfish processed to be eaten may be high (75–124 mg/kg, [76]) or extremely high (400–1800 mg/kg, https://www.cfs.gov.hk/english/programme/programme_rafs/files/Guidelines_on_the_use_of_Al_additives_e.pdf, accessed on 10 April 2022). Regardless of the exact content, high levels of aluminium have been shown to potentially cause memory and cognitive disorders, which may result in neurodegenerative processes such as Alzheimer’s and Parkinson’s diseases [77,78,79,80]. Very recently, a new protocol using calcium lactate (E327), calcium citrate (E333) and calcium acetate (E263) has been proposed as an alternative to aluminium salt-based processing [81]. Considering that processed jellyfish have similar characteristics to those processed using aluminium salts, and the agents used in the recently proposed protocol are approved by the European Community, calcium-based agents may favour the exploitation of jellyfish as a food in EU countries.

Although some studies are suggesting the direct consumption of jellyfish as a food in western countries [66], the introduction of jellyfish into the western dietary composition may find resistance from local populations who are unused to consuming this specific marine organism directly. Yet, the benefits may be achieved by introducing jellyfish into the human body through nutraceutical products such as integrators. A search on the web has highlighted that an integrator based on collagen from scyphomedusae is already in commerce and has received Federal and Drug Administration (FDA) approval in the United States (https://www.longevitybynature.biz/product/kollajell/e, accessed on 10 April 2022). It is likely that people resistant to eating jellyfish directly are willing to ingest them in the form of pills to obtain their beneficial effects.

### 2.2. Biomedicals

#### 2.2.1. Green Fluorescent Proteins (GFPs)

Several marine organisms are bioluminescent. While the chemical mechanisms which lead to the production of light pulses have been defined in some detail, the evolutionary and ecological functions of light emission in the marine environment are not completely clear yet, and range from partner attraction to a defense from potential predators [82]. Among marine animals, jellyfish include several bioluminescent species already known and many potentially unknown, particularly those living in deep waters. The hydromedusa *Aequorea victoria* is likely one of the most famous bioluminescent jellyfish for its Green Fluorescent Protein (GFP), which was first extracted in 1962 by O. Shimomura, M. Chalfie and R. Tsien, who were awarded the Nobel Prize in Chemistry in 2008. Other similar proteins were identified in time and grouped with the first under the label GFPs. They have found a large application in the biomedical field for tagging cells in oncology and nerve cell development. Considering that almost all ctenophores produce light as well as many hydro- and scyphomedusae, such as the mauve stinger, *Pelagia noctiluca*, which often blooms in the Mediterranean Sea and UK waters [25,83], GFPs are likely a potential compound for which jellyfish by-catches may be exploited.

#### 2.2.2. Collagen

As mentioned above, jellyfish are produced mainly by proteins (Table 1), which are organized into a complex polymer, collagen. Collagen, with its fibrils, is the bulk component of most jellyfish (Table 3).

As discussed above for the biochemical composition, the determination of the collagen content is performed on dry or wet mass, which makes a remarkable difference considering that jellyfish, particularly scyphomedusae, contain >90% of water [91]. As a consequence, determinations of the collagen content made on dry mass are at least an order of magnitude greater than determinations made on wet mass (Table 3). Additional differences are due to diverse extraction protocols. The most used protocols are based either on acid or pepsin, with pepsin solubilization being the most effective to maximize the yield of collagen, based on a direct comparison of the two methods [43,85]. Regardless of these differences, the edible scyphomedusa *Rhopilema asamushi* and *Stomolophus meleagris* have the highest content of collagen within the scyphomedusae where it was determined (Table 3).

As suggested in previous reviews [65,92], collagen extracted from jellyfish, particularly scyphomedusae, may be used for biomaterials such as wound-healing and tissue-regenerating items (for example, bandages) [93,94]. However, the high value of this com-pound, due to its biocompatibility with human collagen, convincingly supports its use for biomedical applications, particularly two for which the number of available studies is growing fast:

- Scaffolds. The biocompatibility between human and collagen extracted from scyphomedusae was determined about 20 years ago [95]. In this pilot study, the authors suggested the use of jellyfish collagen for scaffolds to stimulate tissue regeneration and monitored the inflammatory and immune responses to the implantation. The results encouraged the use of jelly-derived scaffolds, which found further support in following studies. The collagens extracted from *Rhopilema esculentum* and *Nemopilema nomurai* were used to design porous scaffolds for cartilage regeneration [96,97]. More recently, biphasic monolithic scaffolds made of jellyfish collagens were shown to be suitable for osteochondral engineering [98]. Jelly-derived collagen tubular scaffolds, modeled as vascular grafts, enhanced vascular endothelial cell development and its mechanical strength [99].
- Drug delivery. After marketing the collagen extracted from the scyphomedusa *Rhizostoma octopus*, a recent study funded by the Jellagen© company which produces it indicated that jellyfish collagen is a suitable cell matrix to culture human-induced pluripotent stem cell-derived Microglia (iMGL) that possess the morphological, surface marker expression and functional characteristics required for microglia. Jellyfish-extracted collagen showed a biological impact on human cells higher than mammalian type I collagen extracted from rat tails. Comparisons were performed by testing adhesion, cell viability and immunocytochemistry assays. These results suggest that collagen from *R. octopus* is a potential inert, non-reactive biomaterial suitable as a substitute for the collagen extracted from rat tail, since cells cultured on this substrate produced significant clumping and cell death [100]. Although more tests are needed to define the suitability of jellyfish collagen compared to other substrates, the fact that microglia play crucial roles within the central nervous system by ensuring synaptic plasticity, immune activity, neurogenesis and homeostasis, the potential application of jellyfish collagen may benefit the study of neural transmission and improve the treatment of diseases resulting from the degeneration of neural networks, such as Alzheimer’s disease.

In line with this study, the collagen extracted from another rhizostome scyphomedusa, *Catostylus tagi*, has been tested to produce microparticles for the controlled delivery of therapeutic proteins [101].

#### 2.2.3. Crude Venom

Toxins contained in the venom injected by the nematocysts of pelagic cnidarians have been studied in greater detail in species belonging to cubomedusae and scyphomedusae compared to hydromedusae because they sting swimmers and sometimes have a lethal effect [102,103]. Overall, jellyfish venoms have negative effects on humans, including cytolytic, cytotoxic, hemolytic, neurotoxic and cardiotoxic effects among the most common activities [103,104,105]. However, some of these negative effects may be used to benefit human health, as suggested by an early study [106] and supported by more recent studies, as summarized below.

The venom from the cubomedusa *Chironex fleckeri* has a lethal effect on humans due to its toxins. However, these effects may be re-directed toward beneficial cardiovascular and cytolytic applications [104]. A growing number of studies determined the suitability of scyphozoan venoms to be used as anti-cancer drugs. The venom extracted from *Nemopilema nomurai* was tested on heart and muscle myoblasts in mice and blood cells from different organisms, including humans [105] and a model animal [107], as well as human hepatocellular carcinoma (HepG2) cells [108]. The crude venom of *Cyanea nozaki* resulted in negatively affecting colon cancer and hepatoma cells in humans [109]. The crude venom of *Pelagia noctiluca* had cytotoxic and cytolytic effects on lung fibroblasts in Chinese hamsters, colon cancer in humans [110], glioblastoma in humans [111] and kidney cells [112]. *Rhizostoma pulmo* induces mild stings to humans, but its crude venom was found to be cytotoxic for lung fibroblasts in Chinese hamsters [113]. Collectively, these studies support the suitability of venom extracted from jellyfish for developing anti-cancer drugs.

### 2.3. Biomaterials

Within jellyfish, scyphomedusae are known to secrete large amounts of mucus [91]. Often the water where they have been detected remains stinging after they have moved somewhere else, likely because mucus is embedded with the stinging cells ejected with it [114]. Stressful conditions stimulate the production of mucus, which appears to be a defense of the animal, considering the presence of stinging cells within it [115]. Mucus, which is considered an additional nuisance associated with jellyfish, particularly during outbreaks, has been recently re-evaluated. Mucus is highly similar to the scyphomedusa which produces it, from a biochemical point of view [116]. From an ecological perspective, mucus, like jelly-falls, physically transfers organic matter into biogeochemical cycles [116]. From the biotechnological point of view, recent studies have proposed mucus as a trap for different types of nanoplastics polluting the sea [117,118]. A laboratory study started to explore the concentration of mucus and their efficiency to sequester nanoparticles in the water [118]. Although these results are preliminary, this use of a by-product of jellyfish appears to be a potential benefit for the marine environment and society, considering the increasing concern due to plastic pollution at sea.

## 3. Conclusions

Jellyfish are actively fished in some areas of the world and processed for food consumption. However, their use may be extended beyond food. In this review, we indicate the potential applications of jellyfish in biotechnology, particularly nutraceuticals, biomedicals and biomaterials, based on the most advanced and promising research studies available in the literature. These studies collectively suggest that jellyfish are a resource more than a nuisance and may provide socio-economic benefits. At present, this potential is highly underestimated and underexploited. Conversely, jellyfish may become a sustainable resource by collecting and processing the fisheries by-catches where jellyfish may often be abundant. By reviewing the main and most promising applications of the compounds extracted from jellyfish, we suggest that jellyfish by-catches are at present a waste-product of fisheries activities to be disposed of, while they need to be re-evaluated as an important source of high-value compounds within the context of a circular and sustainable economy. Like all resources, jellyfish by-catches will need effective management to maximize exploitation, despite the potential limitations due to fluctuating and variable biomass availability. However, the challenge is balanced by the high value of compounds extracted from jellyfish and their potential applications.

## Figures and Tables

**Table 1:** Biochemical composition of jellyfish (hydromedusae, scyphomedusae and cubomedusae). Data are percentages of dry (DM) or wet mass (WM) in the whole (W) or different body parts (B, bell; OA, oral arms: G, gonads).

Species	Tissue	Proteins	Lipids	Carbohydrates	Proteins	Lipids	Carbohydrates	Reference
		(% of DM)			(% of WM)			
**Hydromedusae**								
*Aequorea victoria*	W	6.6	2.2	0.7		0.06		[29]
*Aglantha digitale*	W	21.6–22.1	6.0–6.9	0.4–0.9				[29]
	W	56.5	3.0	0.8				[30]
*Botrynema brucei*	W	7.4 *	1.8 *	0.4 *	0.3 ± 0.03	0.08 ± 0.03	0.02 ± 0.01	[31]
*Bougainvillia superciliaris*	W	7.7–14.9	6.8–10.0	0.7–1.0				[29]
*Calycopsis borchgrevinki*	W		3.1			0.1		[32]
	W	11.2 *	2.2 *	1.1 *	0.5 ± 0.1	0.1 ± 0.03	0.05 ± 0.01	[31]
*Dimophyes arctica*	W		5.8			0.3		[32]
*Diphyes antarctica*	W		1.3 ± 0.3			0.07 ± 0.02		[32]
	W	13.4 *	3.2 *	1.2 *	0.6 ± 0.04	0.1 ± 0.05	0.06 ± 0.01	[31]
*Halitholus cirratus*	W	10.4–18.2	4.6–7.6	0.7–0.8				[29]
*Hybocodon polifer*	W	23.0–31.0	13.1–22.1	0.8				[29]
*Olindias sambaquiensis*	W	14.2 ± 0.02	1.6 ± 0.4	4.7 ± 0.2				[33]
*Rhacostoma atlanticum*	W	10.5 ± 0.01	1.4 ± 0.1	1.2 ± 0.1				[33]
*Sarsia princeps*	W	14.5–14.7	7.8–9.1	0.4–0.8				[29]
**Scyphomedusae**								
Semaeostomeae								
*Aurelia aurita*	W		0.5			0.0		[34]
	W		0.4					[35]
	W		0.2					[36]
	W	4.7	9.2	13.5	5.3	2.0	3.4	[37]
	W	5.9	1.9	2.9				[38]
	G	23.7		14.6				[38]
	OA	7.3		2.6				[38]
*Aurelia aurita*	B	4.2		1.5				[38]
	W	2.1–28.6	1.2–3.4	0.4–1.1				[39]
	G	4.4–23.0	2.6–6.0	1.1–2.1				[39]
	OA	4.1–15.3	1.3–4.0	0.6–1.5				[39]
	B	2.3–8.3	0.9–2.9	0.3–0.9				[39]
	W		0.7			0.03-0.04		[40]
	W	3.5	0.4	19.9				[41]
*Aurelia coerulea*	W				0.25			[42]
	B				0.11			[42]
	OA				0.18			[42]
*Aurelia* sp.1	W	5.7	4.1 ± 0.5					[43]
*Chrysaora hysocella*	W		2.7					[36]
	W	4.6 ± 2.8	1.5 ± 2.1	0.8 ± 2.2	0.2 ± 0.1	0.04 ± 0.03	0.01 ± 0.01	[44]
	G	12.8 ± 6.8	4.5 ± 2.8	0.6 ± 0.04				[44]
	B	3.5 ± 2.6	0.6 ± 0.6	0.1 ± 0.08				[44]
*Chrysaora lactea*	W	12.6 ± 0.01	1.8 ± 1.1	0.9 ± 0.1				[33]
*Chrysaora pacifica*	W	7.5	0.7	22.7				[41]
*Chrysaora quinquecirrha*	W					0.2		[34]
*Cyanea capillata*	G	28.4 ± 3.9	0.6	0.9				[45]
	OA	29.8 ± 3.1	1.2 ± 0.6	1.0 ± 0.1				[45]
	B	7.9 ± 1.5	0.2 ± 0.1	0.8 ± 0.1				[45]
	W	16.5 ± 3.0	0.5 ± 0.1	0.9 ± 0.02				[45]
	G	9.6	1.6	1.0				[46]
	W		0.3–0.8					[47]
*Cyanea lamarcki*	W		0.7					[36]
*Pelagia noctiluca*	W	10.9–19.8	1.3–2.9	0.1–0.7				[48,49]
	W					0.2		[50]
*Poralia rufescens*	W	0.2	0.4	0.1				[46]
*Stygiomedusa gigantea*	W		10.2			0.5		[32]
Rhizostomeae								
*Acromitus maculosus*	B	21.4 ± 0.3	0.4 ± 0.2	17.7	0.8 ± 1.2			[51]
*(A. hardenbergi)*	OA	33.7 ± 1.1	1.1 ± 0.2	6.0	1.3 ± 1.0			[51]
*Cassiopea andromeda*	W		0.9			0.07		[52]
*Catostylus tagi*	W		0.8 *			0.4		[53]
	B	8.4 *	1.0 *		1.8	0.2		[53]
	OA	18.0 *	2.2 *		4.3	0.5		[53]
*Cotylorhiza tuberculata*	W	2.2	12.3 ± 0.7					[43]
	B	7.6–12.0	0.5–0.7					[54]
	OA	20.0	6.4					[54]
	G	36.8	6.0					[54]
*Lychnorhiza lucerna*	W	12.3 ± 0.03		2.7 ± 0.04				[33]
*Rhizostoma luteum*	W	0.8–1.9						[55]
*Rhizostoma octopus*	G	12.1 ± 9.8	0.6 ± 0.4	0.9 ± 0.03				[45]
	OA	13.4 ± 0.4	0.3 ± 0.1	0.7 ± 0.3				[45]
	B	6.6 ± 2.3	0.3 ± 0.1	0.7 ± 0.01				[45]
	W	12.8 ± 2.3	0.3	0.8				[45]
*Rhizostoma pulmo*	W		2.3					[56]
	W	6.0	4.0 ± 0.1					[43]
	B	8.7–13.7	0.7–1.0					[54]
	OA	27.0	0.8					[54]
	G	18.0	1.2					[54]
*Rhopilema hispidum*	B	19.9 ± 0.7	0.5 ± 0.3	18.2	0.5 ± 0.2			[51]
	OA	43.8 ± 0.2	1.4 ± 0.2	10.7	2.0 ± 1.6			[51]
*Rhopilema esculentum*	B	38.1 ± 1.1	0.6 ± 0.1	8.9	1.6 ± 0.8			[51]
	OA	53.9 ± 2.1	1.8 ± 0.3	7.7	2.7 ± 0.9			[51]
*Stomolophus meleagris*	B				1.1 ± 0.2			[57]
	B				1.0 ± 0.1			[57]
Coronatae								
*Atolla wyvillei*	W		1.1					[58]
	W	16.9 *	4.2 *	1.7 *	0.8 ± 0.3	0.2 ± 0.1	0.1 ± 0.01	[31]
	W		0.3			0.01		[32]
*Periphylla periphylla*	W	6.4 ± 1.7	2.1 ± 0.8	0.9 ± 0.2	0.3 ± 0.1	0.1 ± 0.06	0.05 ± 0.02	[59]
**Cubomedusae**								
*Chiropsalmus quadrumanus*	W	18.2 ± 0.02	1.3 ± 0.0	5.9 ± 0.3				[33]
*Tamoya haplonema*	W	27.7 ± 0.03	3.7 ± 0.4	4.2 ± 0.0				[33]

* Calculated by the authors from percentage of WM/total DM.

**Table 2:** Content (% of total fatty acids) of fatty acids most relevant to human health in diverse species of jellyfish (hydromedusae and scyphomedusae).

Species	Fatty Acids																								Reference
	12:0	14:0	15:0	16:0	17:0	18:0	20:0	16:1(n-7)	18:1(n-9)	18:1(n-7)	20:1(n-9)	20:1(n-7)	22:1(n-11)	22:1(n-9)	18:2(n-6)	18:3(n-3)	18:4(n-3)	20:4(n-6)	20:4(n-3)	20:5(n-3)	22:4(n-6)	22:5(n-6)	22:5(n-3)	22:6(n-3)	
**Hydromedusae**																									
*Aequorea victoria*		1.7	0.8	7.7	0.9	8.9	1.0	2.6	5.4	1.1	2.1	3.1		6.2	0.4	0.3	0.4	9.6	0.4	9.6	0.3	4.4	1.5	18.8	[67]
*Arctapodema ampla*	0.3	5.5	1.2	14.9	0.5	5.1	0.3	2.8	5.7	2.0	1.8	2.0		2.7	0.9	0.1	0.2		0.3	16.4			0.2	26.4	[68]
*Calycopsis borchgrevinki*	0.5	2.5	0.3	16.6	0.0	4.8		9.2	26.4	3.7	2.5	2.8	0.0	0.0	1.5	0.2	0.1	1.4		9.6			2.1	7.0	[32]
	0.1	2.7	0.6	11.6	0.6	7.8	0.2	5.2	14.3	1.9	4.4	6.9	0.9	0.3	0.9		0.1		0.8	9.9	2.1		5.6	16.1	[68]
*Chelophyes appendiculata*		2.4	0.0	11.0	0.0	7.6		6.6	11.9									4.3		8.1				19.2	[69]
*Dimophyes arctica*	0.0	3.9	0.8	18.9	0.0	9.3		15.3	17.2	4.1	1.1	1.0	0.0	1.3	1.0	0.1	0.2	0.0		8.2			0.1	10.4	[32]
*Diphyes antarctica*	0.0	7.6	1.1	18.0	0.0	8.8		4.0	8.7	2.0	1.2	3.6	0.2	0.1	2.2	0.3	2.1	1.4		16.5			0.2	16.9	[32]
	0.8	5.7	1.0	20.1	0.0	7.6		3.8	7.3	4.1	1.7	0.6	0.0	0.0	1.9	0.2	0.6	1.2		19.1			0.3	17.6	[32]
**Scyphomedusae**																									
*Atolla wyvillei*	0.6	6.1	0.6	20.8	0.0	2.8	0.0	5.3	11.8	7.2	3.2		0.2	0.0	2.5	1.0	5.0		0.0	16.6	0.3		2.4	5.6	[32]
*Aurelia aurita*		1.5	0.4	12.0	1.1	10.0	0.1	0.2	0.9	1.8	0.4	0.2	0.4		0.9	1.0	1.3	1.8	0.9	33.3	0.3	0.1	5.0	11.2	[70]
	1.2	4.1	2.8	42.3	2.9	22.2	0.0	2.6	8.8						1.8			1.4		0.7					[40]
		2.2		13.7		7.4		1.0	3.3									9.7		3.3				6.8	[69]
	0.1	3.8	1.5	23.1	1.2	21.9	0.9	5.1	4.0	1.6	0.8		2.3		1.3	0.6	0.8	4.5		17.6			2.2	5.1	[36]
	0.0	3.3	1.0	16.0	0.7	6.4	1.2	4.6	8.9	2.6	4.8	7.1	1.2	3.2	0.8	0.4	0.1	6.7	0.4	8.5	0.6	0.3	1.6	7.0	[71]
		3.2	1.0	11.5	0.5	13.3	0.3	3.1	4.4	1.3					0.9	0.4		15.2		19.4	1.7	0.5	5.8	12.8	[72]
	0.4	2.8	2.1	22.5	5.4	19.2	0.3	4.5	6.2		0.6			0.1	1.3	0.9	1.9		7.8	17.5				1.3	[41]
		2.9	1.0	24.3	1.8	15.7	0.3	4.7	2.2	2.3	0.4			8.2	1.0	0.7	0.4	4.8		12.7			1.9	5.2	[73]
*Aurelia* sp. 1	0.0	2.4		33.0	1.4	32.7	0.0	0.0	3.0	1.7					1.3			5.5	0.0	14.6				4.4	[43]
*Cassiopea andromeda*	9.3	4.2		21.9		12.5	0.6	4.3	2.8						0.8	2.6	7.4	14.2		2.1	2.9		2.5	11.0	[52]
*Chrysaora isoceles*	0.0	3.3	0.4	9.5	0.6	7.1	1.2	3.7	4.4	1.5	6.6		6.1		0.8	0.7	1.3	5.4		20.0			5.4	19.7	[36]
*Chrysaora pacifica*	0.4	3.7	2.7	14.9	4.5	18.1	0.6	5.9	5.1		1.0			0.4	0.7	0.8	1.7		13.5	15.2				3.2	[41]
*Chrysaora quinquecirrha*		0.7		10.0		10.9	1.9	3.2							0.5	0.4	0.1	22.4		7.6	6.3		3.6	11.9	[34]
*Cotylorhiza tuberculata*	0.0	2.9		26.1	0.8	24.2	0.8	1.2	12.8	1.2					8.3			5.3	4.1	5.1				7.2	[43]
*Cyanea capillata*		1.7	1.0	12.9	1.9	6.1	2.0	4.0	6.2		10.5			2.1	0.4	0.5	0.4	7.8	0.6	15.0	1.3	1.1	3.7	14.4	[47]
*Cyanea lamarcki*	0.1	3.5	1.3	19.0	1.0	12.0	1.8	4.7	5.5	3.1	3.4		2.5		0.4	0.4	0.6	8.7		13.8			3.2	12.1	[36]
*Cyanea nozakii*		2.3	0.9	11.1	0.9	13.9	1.3	3.2	2.9	1.3					1.0	0.4		22.9		11.9	4.7	1.6	4.6	9.9	[72]
*Pelagia noctiluca*		0.2	0.2	69.2	0.9	15.2	0.0	0.0	0.2	0.1	0.0	0.0		0.1	0.0	0.0	0.0	0.6		6.2	0.0	0.0	0.3	2.3	[18]
		3.0	2.4	33.2	4.9	18.0	0.6	2.1	5.7	4.2	1.6		2.2	2.7	0.9	0.5	0.1	0.8	0.1	1.1	0.1	0.4	0.7	0.7	[74]
*Periphylla periphylla*	0.0	3.8	0.5	15.7	0.5	7.8	0.2	2.4	15.5	3.2	4.4	5.1	1.7	3.3	1.0	0.2	0.8		0.7	20.9			3.4	0.5	[68]
	0.0	1.0	0.2	13.5	0.8	21.5	0.6	0.0	6.7	2.4	4.5	4.7	2.4	4.5	0.3			0.3		1.5			1.0	19.4	[68]
	0.1	3.1	0.5	13.6	0.4	7.0	0.2	2.1	14.3	2.5	4.7	5.2	1.9	3.2	0.9	0.1	0.6		0.6	17.7			2.6	12.1	[68]
*Rhizostoma luteum*	0.3	2.0	0.4	11.0	0.7	15.0	0.1	3.1	11.3		1.0			0.1	4.6	9.8		23.7		3.6				0.3	[55]
*Rhizostoma octopus*	0.3	5.1	2.0	27.3	2.3	21.7	0.0	3.8	6.8	3.5	0.8				1.6	1.6	2.8	2.8		9.7			1.3	5.3	[36]
*Rhizostoma pulmo*	1.3	3.1		33.2	0.0	30.6	0.0		5.1	1.9					2.5			8.8	0.0	8.6				4.9	[43]
*Rhopilema esculentum*		1.5	0.5	12.5	1.3	12.6	0.5	2.0	2.0	2.7	0.3	0.2			1.8	1.4	2.4	8.4		13.1	2.4	1.0	5.1	12.3	[70]
*Stomolophus meleagris*		1.7		12.1		8.7	0.5	2.9					0.1		0.9	0.5	1.1	15.5		19.1	2.6	1.2	3.1	15.9	[34]
*Stygiomedusa gigantea*	0.0	6.6	0.6	12.1	0.0	4.9	0.0	10.2	18.6	5.8	2.6		0.1	0.3	1.0	0.3	1.3	0.0	0.0	20.8	0.7		1.9	4.2	[32]

**Table 3:** Collagen content (percentage of dry (DM) or wet mass (WM)) in different species of scyphomedusae extracted using two different protocols based either on acid or pepsin solubilization. B, bell; OA, oral arms; W, whole; M, mesoglea.

Species	Tissue	Collagen Content				Reference
		Pepsin		Acid		
		(% DM)	(% WM)	(% DM)	(% WM)	
*Aurelia aurita*	W		0.01			[84]
*Chrysaora* sp.	B		9–19			[85]
*Pelagia noctiluca*	W		0.07			[84]
*Cassiopea andromeda*	W	2.2–6.0				[52]
*Catostylus tagi*	B	2.7				[86]
*Cotylorhiza tuberculata*	B		4.5			[84]
	OA		19.4			[84]
	B	<10				[84]
*Rhizostoma pulmo*	B		8.3–31.5			[84]
	OA		26–90			[84]
	B	<10				[84]
*Rhopilema asamushi*	M	35.2				[87]
*Rhopilema esculentum*	M		0.28		0.12	[88]
*Stomolophus meleagris*	M	46.4				[89]
*Nemopilema nomurai*	M	2.2				[90]

## Data Availability

Not applicable.
